# Long-Term Efficacy of Postpartum Intravenous Iron Therapy

**DOI:** 10.1155/2014/815437

**Published:** 2014-11-06

**Authors:** Nadine Becuzzi, Roland Zimmermann, Alexander Krafft

**Affiliations:** Division of Obstetrics, Department of Obstetrics and Gynecology, University Hospital Zurich, 8091 Zurich, Switzerland

## Abstract

*Background*. The potential benefits of administering a dose of intravenous iron in patients with moderate postpartum anaemia rather than oral iron alone remains unproven. *Aims.* To determine whether a single injection of intravenous iron followed by a 6-week course of oral iron is as effective over 6 months in restoring normal haemoglobin levels and replenishing iron stores in women with moderate postpartum anaemia as a course of oral iron alone in women with mild postpartum anaemia. *Materials and Methods*. Retrospective two-arm cohort study in women with mild postpartum anaemia (haemoglobin 9.6–10.5 g/dL) prescribed iron daily for 6 weeks (*N* = 150) and women with moderate postpartum anaemia (haemoglobin 8.5–9.5 g/dL), given a single 500 mg injection of intravenous iron followed by iron daily for 6 weeks (*N* = 75). Haemoglobin and ferritin were measured 6 months postpartum. *Results*. Haemoglobin returned to similar mean levels in both groups. Ferritin levels were statistically significantly higher in the intravenous + oral group (57.7 ± 49.3 *μ*g/L versus 32.9 ± 20.1 *μ*g/L). *Conclusions.* Despite lower baseline haemoglobin, intravenous iron carboxymaltose was superior to oral iron alone in replenishing iron stores in moderate postpartum anaemia and may prove similarly beneficial in mild postpartum anaemia.

## 1. Introduction

Postpartum anaemia, defined by the World Health Organization as a haemoglobin (Hb) level < 11.0 g/dL, is a very common obstetric problem usually resulting from a combination of blood loss at delivery and preexisting iron deficiency [1]. Its reported incidence 6 months postpartum ranges from 12.7% to 30% [2–4]. Symptoms include lactation failure and postpartum depression [3–5]. Stepwise interventions commonly tailor treatment to the postpartum Hb level, using oral iron for mild cases and intravenous iron for moderate to severe cases, although cut-offs vary considerably [6, 7].

We earlier demonstrated the efficacy of oral iron in postpartum iron deficiency without anaemia [8]. However, several studies have since shown that intravenous therapy is more effective than its oral counterpart for replenishing iron stores in postpartum anaemia [9–12].

In the present retrospective study we postulated that women treated with oral iron only for mild postpartum anaemia would have lower ferritin levels at 6 months than those treated with intravenous + oral iron for more severe anaemia. Our aim was to compare efficacy at 6 months between treating mild postpartum anaemia with oral iron versus moderate anaemia with intravenous + oral iron under the conditions of our institution's stepwise management schedule modelled on current best-practice recommendations [13].

## 2. Methods

The institution's ethics committee approved the study protocol. Women were informed about the study by post around 5 months postpartum and all gave their informed consent prior to participation. Data of patients were obtained from the hospitals patients records.

Primary endpoints of the study were Hb and ferritin at 6 months postpartum (±28 days). The following parameters were measured: whole blood count (flow cytometry: Advia 120, Bayer Health Care, Leverkusen, Germany), ferritin (sandwich immunoassay: Automated Chemiluminescence System 180, Chiron Diagnostics, East Walpole, MA, USA), and C-reactive protein (CRP; turbidimetric immunoassay: TinaQuant, Roche Diagnostics, Rotkreuz, Switzerland).

All women had been prescribed oral iron sulphate containing 80 mg elemental iron/day alone for 6 weeks after discharge for those with Hb 9.6–10.5 g/dL measured 24–48 h post-partum (*n* = 150) or in the case of post-partum Hb 8.5–9.5 g/dL (*n* = 77) they had received a single infusion of 500 mg iron carboxymaltose (Ferinject) diluted in 100 mL sodium chloride 0.9% followed by the same prescription for oral iron sulphate containing 80 mg elemental iron/day.

Patients were questioned 6 months postpartum about oral medication compliance, physical performance (conventional Hayes and Paterson visual analogue scale (VAS) from 1 to 10 comparable to a VAS for pain), diet (vegetarian/nonvegetarian), duration of breast-feeding, degree of menstrual blood loss, and adverse events during intravenous and oral iron therapy. Exclusion criteria were surgery, blood transfusion, and/or additional intravenous iron therapy within the intervening 6 months.

### 2.1. Statistical Analysis

Groups were compared using the Mann-Whitney test or Pearson's chi-squared test where appropriate. A *P* value < 0.05 was used in all tests. Data are given as the mean ± SD. Sample sizes were calculated by power analysis (StatView 5.0.1, SAS Institute, Cary, NC, USA).

## 3. Results

### 3.1. Patient Characteristics

After reviewing the records of 1509 women with postpartum anaemia over a 26-month period, we included 225 women in the study. Three women were administered an additional dose of intravenous iron postpartum and were therefore excluded. None underwent surgery or received blood transfusions in the 6 months postpartum. The remaining 1281 women either could not be reached or decided not to participate. One hundred and fifty women had postpartum Hb 9.6–10.5 g/dL and were assigned to oral iron only; 75 women had postpartum Hb 8.5–9.5 g/dL and received intravenous + oral iron. Maternal age at delivery, gestational age at delivery, number of previous pregnancies, and number of previous births did not differ significantly between the two groups (Table 1).

### 3.2. Haematological Response (Table 2)

At 6 months only three and four women remained anaemic in the oral and intravenous + oral groups. Mean Hb increased from 10.1 g/dL postpartum to 13.3 ± 0.83 g/dL at 6 months versus from 9.1 g/dL to 13.3 ± 0.84 g/dL (*P* = 0.68). Of all the other haematological parameters, only mean corpuscular haemoglobin significantly differed statistically between the groups, being higher in the intravenous + oral group (*P* < 0.05).

### 3.3. Iron Status and Inflammatory Response

Mean ferritin at 6 months was statistically significantly lower in the oral group: 32.9 ± 20.1 *μ*g/L (range 4–111 *μ*g/L) versus 57.7 ± 49.3 *μ*g/L (range 9–296 *μ*g/L) (*P* < 0.0001).

CRP levels were similar in both groups: 2.0 ± 4.4 mg/L versus 1.8 ± 2.7 mg/L.

### 3.4. Questionnaire Results (Table 3)

Self-reported durations of oral iron supplementation ranged from 0 to 180 days across groups, with a median of 46 days in the intravenous + oral group and 30 days in the oral iron group, respectively; 52% of patients in intravenous + oral group and 20% of patients in the oral iron group took no oral treatment at all.

There were no statistical significant intergroup differences in breast-feeding practice and duration, proportion of vegetarians, self-rated physical performance, or reported return of menstruation at 6 months (71.6% in the oral iron group and 62.7% in the intravenous + oral group).

### 3.5. Adverse Events

No intravenous iron recipient could recall any adverse effects, although one woman elected not to participate due to persistent cutaneous infiltration after injection-related extravasation. While the intravenous administration was generally very well tolerated, the women in the orally supplemented group frequently complained of gastrointestinal symptoms, such as abdominal pain and constipation.

## 4. Discussion

Our results show that iron supplementation, whether oral only or intravenous + oral, is an effective treatment of postpartum anaemia. Women with initially more severe anaemia treated with intravenous + oral iron reached the same Hb level 6 months later as those prescribed oral iron for mild anaemia. However, our data show that intravenous supplementation is the more effective approach for long-term iron store replenishment. Safety and tolerability studies of iron carboxymaltose show infrequent side effects [14–16].

Previous studies found oral iron and intravenous iron to be equieffective treatments for correcting postpartum anaemic Hb levels up to the initial three months [9, 11]. The novelty of our study is that we measured laboratory parameters at 6 months, thus giving a more accurate estimate of long-term effect.

Despite being anaemic after delivery, all but seven patients (four and three, resp.) had normal Hb levels at 6 months. The lowest Hb at 6 months was 10.9 g/dL. One patient in the intravenous + oral group with an Hb of 11.2 g/dL combined with a ferritin of 296 *μ*g/L was referred to a haematologist for a possible haemoglobinopathy.

Mean ferritin levels were statistically significantly higher in the intravenous + oral group: although these women had been more anaemic on starting treatment, their iron stores were better restored half a year later. One reason might be the well-documented problems associated with the intestinal absorption of oral iron, including iron sulphate. Various factors such as diet may impair oral iron uptake. Postpartum serum ferritin levels were not measured given that ferritin can be elevated as an acute-phase protein for at least the first two weeks of the puerperium, thus showing no correlation with iron stores [17].

Gastrointestinal side effects are a well-recognised contributor to poor patient compliance with long-term oral iron treatment [18]. Many of the women in our study who ended oral iron supplementation shortly after discharge complained of abdominal pain and constipation. Twenty percent in the oral iron alone group and 52% of women in the intravenous + oral group did not even start oral treatment. One might speculate that the statistical significant higher rate of nonstarters of oral iron in intravenous + oral group is based on a lesser psychological strain after having had an iron infusion. However, the remaining women who continued oral treatment must be presumed to have done so, based on their self-reported declarations, for considerably longer than the prescribed 6 weeks (42 days) in order to have achieved a median duration of oral iron supplementation of 46 days. The compliance figures in these women were higher than expected, bearing in mind that they were not being regularly encouraged to take the tablets.

The fact that we administered the questionnaire retrospectively, 4.5 months after the nominal completion of the 6 week oral therapy, could account for inaccurate recollection. We conclude from the apparent spread of self-reported compliance data and the absence of any external compliance monitoring that our data simply reflect the noninterventional observational nature of our study. They are the findings to be expected under real-life practice conditions, representing actual outcomes in unobserved patients in contrast to data obtained in studies of closely supervised patients.

Our findings are consistent with those of Bhandal and Russell who showed that intravenous iron sucrose increases Hb levels more rapidly and replenishes iron stores more efficiently than oral iron sulphate [9]. On postpartum day 40 Hb levels in both their study groups were similar, but iron reserves were significantly higher in the intravenous group. Our study now shows that this difference remains statistically significant for up to 6 months. However, in contrast to our own population, the women in the Bhandal and Russell study were all severely anaemic, with similarly low mean group Hb levels on starting treatment (7.5 g/dL and 7.3 g/dL). We also prescribed a lower dose of oral iron sulphate than Bhandal and Russell in order to maintain tolerability while remaining effective in restoring the depleted iron stores [19].

Given that postpartum injection of intravenous iron carboxymaltose is more effective than oral alternatives in replenishing iron resources, we would advocate its use as the treatment of choice not only in moderately severe anaemia but also in mild postpartum anaemia, in particular for women planning a subsequent pregnancy. However, further studies are needed to determine the precise impact of postpartum anaemia therapy on a future pregnancy.

## Figures and Tables

**Table 1:** Baseline data and characteristics.

Characteristics	Oral iron	Intravenous + oral iron	*P*
	(*N* = 150)	(*N* = 75)	
Maternal age at delivery (years)	32.1 (5.4)	31.5 (6.1)	0.46
Gestational age (weeks)	39.2 (2.9)	39.1 (3.0)	0.91
Gravidity	2.0 (1.4)	2.1 (1.6)	0.77
Parity	1.6 (0.9)	1.5 (1.1)	0.63
Blood loss, estimated (mL)	537.2 (283.5)	625.9 (404.9)	0.06
Hb after delivery (g/dL)	10.1 (0.3)[9.5–10.5]	9.1 (0.3)[8.5–9.5]	<0.0001
Haematocrit after delivery (%)	29.5 (1.4)[25.4–36.2]	26.9 (1.7)[23–31.5]	<0.0001
Mode of delivery			
Vaginal spontaneous	54 (36%)	33 (44%)	
Vaginal operative	28 (18.7%)	9 (12%)	
Caesarean section	68 (45.3%)	33 (44%)	

Values are expressed as mean (SD) [range], or numbers and percentages.

**Table 2:** Laboratory data 6 months post-partum.

Characteristics	Oral iron	Intravenous + oral iron	*P*
	(*N* = 150)	(*N* = 75)	
Hb (g/dL)	13.3 (0.8)[10.9–15.3]	13.3 (0.8)[11.1–15.7]	0.68
Haematocrit (%)	39.5 (2.3)[32.8–46.4]	39.4 (2.6)[34.1–47.6]	0.78
MCV (fL)	85.6 (4.4)[71.7–97.8]	86.6 (5.1)[72.7–97.7]	0.13
MCH (pg)	28.8 (1.7)[22.5–32.1]	29.3 (1.9)[21.8–34]	0.047
MCHC (g/dL)	33.6 (1.0)[30.4–35.9]	33.8 (1.1)[30.0–37.3]	0.15
Microcytes (%)	1.5 (1.9)[0.1–13.2]	1.3 (2.1)[0.1–13.9]	0.48
Hypochromic erythrocytes (%)	1.7 (3.6)[0.1–24.9]	1.3 (3.0)[0.1–18.9]	0.43
Ferritin (*μ*g/L)	32.9 (20.1)[4–111]	57.7 (49.3)[9–296]	<0.0001
CRP (mg/L)	2.0 (4.4)[0–39]	1.8 (2.7)[0–11]	0.69

Values are expressed as mean (SD) [range].

**Table 3:** Questionnaire data at 6 months post-partum.

	Oral iron	Intravenous + oral iron	*P*
	(*N* = 150)	(*N* = 75)	
Median duration of 80 mg/d oral iron sulphate supplementation (days) (all patients)	30 (46.8)[0–180]	46 (45.2)[0–180]	.02
Women taking oral iron supplementation [*N* (%)]	120 (80%)	36 (48%)	.006
Breast-feeding women [*N* (%)]	131 (87.3%) (mean 20.9 weeks)	68 (90.7%) (mean 20.7 weeks)	.84
Vegetarians [*N* (%)]	11 (7.4%)	8 (10.8%)	.34
Mean physical performance (scale 1–10)	6.9 (1.9)[2–10]	7.2 (1.7)[2–10]	.36
Menstruation 6 months post-partum			
None	42 (28.4%)	28 (37.3%)	.29
Yes	106 (71.6%)	47 (62.7%)	

Values are expressed as mean (SD) [range], or numbers and percentages.
